# Histopathological and Ultrastructural Evaluation of Buccinator Muscle Alterations in Oral Submucous Fibrosis: A Descriptive Light and Transmission Electron Microscopic Study

**DOI:** 10.7759/cureus.114785

**Published:** 2026-08-19

**Authors:** Nishath Sayed Abdul, Kannan Ranganathan, Uma Devi K Rao, Elizabeth Joshua, Rooban Thavarajah

**Affiliations:** 1 Department of Oral Pathology, Riyadh Elm University, Riyadh, SAU; 2 Department of Oral and Maxillofacial Pathology, Ragas Dental College and Hospital, Chennai, IND

**Keywords:** buccinator muscle, histopathology, muscle degeneration, oral submucous fibrosis, transmission electron microscopy

## Abstract

Introduction: Oral submucous fibrosis (OSMF) is a chronic, progressive, potentially malignant disorder characterized by fibrosis of the oral mucosa and progressive restriction of mouth opening. The present study aimed to descriptively evaluate the histopathological and ultrastructural alterations of the buccinator muscle in patients with OSMF using light microscopy and transmission electron microscopy (TEM).

Materials and methods: This descriptive observational study included 10 buccal mucosal biopsy specimens from 7 patients clinically diagnosed with OSMF and 3 healthy controls. Consecutive sampling was employed, and biopsy specimens measuring approximately 5 × 5 mm, including the underlying buccinator muscle, were collected. One portion of each specimen was processed for hematoxylin and eosin (H&E) staining and examined under a light microscope, whereas the remaining tissue was processed for transmission electron microscopy. Histopathological features, including muscle fiber atrophy, splitting, hyaline degeneration, fibrosis, vascular alterations, and ultrastructural changes involving myofibrils, myofilaments, mitochondria, muscle nuclei, autophagic vacuoles, and collagen deposition, were descriptively documented.

Results: Among the 7 OSMF cases, 2 (28.6%) were clinically classified as grade I, 4 (57.1%) as grade II, and 1 (14.3%) as grade III based on mouth opening. Light microscopic examination revealed muscle fiber atrophy, splitting of muscle fibers, flocculent degeneration, hyaline degeneration, and fibrosis in all OSMF specimens. Constricted blood vessels were observed in 5 (71.4%) specimens, and vacuolar degeneration and dilated blood vessels were identified in 2 (28.6%) specimens each. Transmission electron microscopy revealed loss of myofibrils, loss of myofilaments, focal disruption of I bands and Z lines, irregular Z lines, enlarged mitochondria, enlarged muscle nuclei, autophagic vacuoles, and collagen replacement of muscle fibers in all OSMF specimens. No alterations were observed in the control specimens.

Conclusion: This exploratory descriptive study demonstrated consistent histopathological and ultrastructural degeneration of the buccinator muscle in OSMF. Although the findings provide preliminary evidence of skeletal muscle involvement, they should be interpreted cautiously because of the small sample size, and larger studies are needed to validate these observations.

## Introduction

Oral submucous fibrosis (OSMF) is a chronic, progressive, and potentially malignant disorder characterized by excessive collagen deposition, fibrosis of the oral mucosa, and gradual restriction of mouth opening [1]. A recent systematic review and meta-analysis by Wang et al. [2] reported a pooled global prevalence of 3%, with the highest prevalence observed in India (4%), particularly among individuals with areca nut chewing habits, highlighting the substantial public health burden of OSMF in India. Rani et al. [3] reported a high burden of OSMF among habitual areca nut users and demonstrated a significant association between disease severity and habit-related factors, emphasizing the continuing public health importance of this condition.

OSMF has a well-recognized risk of malignant transformation, making it a significant public health concern. Clinically, patients present with a burning sensation of the oral mucosa, blanching, palpable fibrous bands, progressive trismus, reduced tongue mobility, and difficulty in mastication, swallowing, and speech. Histopathologically, the disease is characterized by epithelial atrophy, juxtaepithelial hyalinization, chronic inflammatory cell infiltration, and progressive fibrosis of connective tissue [4,5].

Although connective tissue fibrosis is considered the hallmark of OSMF, increasing evidence suggests that degeneration of the underlying skeletal muscles also contributes substantially to the progressive limitation of mouth opening [4,6]. The buccinator muscle, which plays an essential role in maintaining oral function, may undergo structural and degenerative alterations secondary to fibrosis or as part of the primary disease process [7]. Conventional light microscopy provides valuable information regarding muscle fiber atrophy, hyalinization, fibrosis, vascular alterations, and inflammation. However, it cannot adequately demonstrate the ultrastructural changes occurring within individual muscle fibers [7].

Transmission electron microscopy (TEM) enables detailed visualization of cellular and subcellular alterations, including disruption of myofibrils, loss of myofilaments, abnormalities of Z lines and I bands, mitochondrial changes, autophagic vacuoles, and collagen deposition surrounding muscle fibers [6]. These ultrastructural findings provide deeper insight into the pathological mechanisms responsible for muscle dysfunction and may help explain the discrepancy between the degree of connective tissue fibrosis and the severity of restricted mouth opening observed in many patients with OSMF [8]. Despite its potential diagnostic value, only a limited number of studies have comprehensively documented muscle alterations using light microscopy and TEM. Therefore, the present exploratory descriptive study was undertaken to document the histopathological and ultrastructural alterations of the buccinator muscle in OSMF using light microscopy and transmission electron microscopy. The study specifically assessed muscle fiber atrophy, fiber splitting, fibrosis, vascular alterations, myofibrillar and myofilament disruption, mitochondrial abnormalities, autophagic vacuoles, and collagen replacement of muscle fibers. Healthy control specimens were included as a descriptive reference for normal muscle morphology. The study was not designed to establish causality or disease mechanisms, or to quantitatively determine associations between these microscopic findings and clinical grade or mouth opening.

## Materials and methods

Study design and setting

This descriptive observational study was conducted at the Department of Oral and Maxillofacial Pathology, Ragas Dental College and Hospital, Chennai, Tamil Nadu, India, between August 2019 and November 2019. Ethical approval was obtained from the Institutional Ethics Committee before the commencement of the study (RDCH/IEC/2019/OMFP/047, dated 18/06/2019), and written informed consent was obtained from all participants prior to biopsy collection.

Study population

The study included 10 buccal mucosal biopsy specimens, comprising 7 consecutive patients clinically diagnosed with OSMF and 3 healthy control subjects who underwent surgical removal of impacted mandibular third molars. Consecutive sampling was employed, whereby all eligible participants who presented during the study period and fulfilled the selection criteria were included. As the objective of the study was descriptive in nature, no formal sample size calculation was performed.

Patients diagnosed with OSMF according to the clinical classification proposed by More et al. [9] were included. Diagnosis was based on a history of areca nut chewing, burning sensation of the oral mucosa, oral mucosa blanching, palpable fibrous bands, and restricted mouth opening. Patients with clinical grades I-III OSMF were eligible for inclusion. Individuals receiving corticosteroids, antifibrotic medications, or any other treatment for OSMF were excluded. The control group comprised systemically healthy individuals without a history of areca nut chewing, tobacco use, smoking, or alcohol consumption, and with no history of oral mucosal disease or restricted mouth opening.

Clinical examination

A detailed case history was obtained from each participant, including demographic information, medical history, dental history, duration and frequency of areca nut use, and associated habits. Clinical examination included assessment of the oral mucosa for blanching, fibrous bands, and other characteristic features of OSMF. Maximum mouth opening was measured as the interincisal distance between the maxillary and mandibular central incisors using a calibrated stainless steel vernier caliper. Based on the measured mouth opening, patients were clinically categorized into grade I (>40 mm), grade II (20-39 mm), and grade III (<20 mm) [10].

Tissue collection

Incisional biopsies measuring approximately 5 × 5 mm were obtained from the buccal mucosa under local anesthesia, ensuring adequate tissue depth to include the underlying buccinator muscle. In the control group, normal buccal mucosa adjacent to the surgical site during impacted third molar removal was harvested using the same dimensions to obtain comparable muscle tissues. Immediately after collection, each biopsy specimen was divided into two equal portions for light microscopic and ultrastructural evaluations.

Light microscopic evaluation

One portion of each biopsy specimen was fixed in 10% neutral buffered formalin, followed by routine tissue processing, paraffin embedding, and sectioning into 4-5 μm-thick sections using a rotary microtome. The sections were stained with hematoxylin and eosin (H&E) and examined using a light microscope (Olympus BX53 research, Olympus Corp., Tokyo, Japan). Histopathological assessment focused on epithelial atrophy, juxtaepithelial hyalinization, degeneration of muscle fibers, muscle fiber atrophy, splitting of muscle fibers, flocculent degeneration, vascular alterations, fibrosis surrounding muscle bundles, and inflammatory changes in the muscle tissue. Representative photomicrographs were captured using an Olympus DP74 digital microscope camera (Olympus Corporation, Tokyo, Japan) mounted on the microscope for documentation purposes.

Transmission electron microscopic evaluation

The remaining tissue specimens were immediately immersed in 2.5% buffered glutaraldehyde for primary fixation. After fixation, the epithelial layer was carefully separated from the connective tissue under a dissecting microscope, and tissue blocks measuring approximately 1 mm³ containing muscle fibers were prepared for ultrastructural examination. The specimens underwent routine TEM processing, including post-fixation, dehydration through graded alcohols, resin embedding, ultrathin sectioning, and staining with uranyl acetate and lead citrate according to the standard laboratory protocols of the Department of Electron Microscopy, Cancer Institute (WIA), Adyar, Chennai, Tamil Nadu, India. Ultrathin sections were examined using a transmission electron microscope (JEOL JEM-1011, JEOL Ltd., Tokyo, Japan), and representative electron micrographs were digitally captured for documentation. Ultrastructural evaluation included the assessment of loss of myofibrils, myofilament disruption, disappearance of I bands and Z lines, irregular Z lines, mitochondrial enlargement, enlarged muscle nuclei, autophagic vacuoles, and collagen deposition replacing muscle fibers.

Data documentation

All histopathological and ultrastructural findings were independently evaluated by two experienced oral pathologists. Prior to the study, the observers were calibrated using representative histopathological and ultrastructural images to standardize the evaluation criteria. All slides and electron micrographs were coded, and the observers were blinded to the clinical diagnosis and grading of the specimens during assessment. Any discrepancies in interpretation were resolved by consensus following joint review. Interobserver reliability was assessed using Cohen’s kappa coefficient. A randomly selected 20% of the histopathological slides and TEM images were independently re-evaluated by both observers for assessment of interobserver reliability. The Cohen kappa values ranged from 0.78 to 0.91 for the key histopathological features (muscle fiber atrophy, splitting of muscle fibers, hyaline degeneration, fibrosis, and vascular alterations) and from 0.80 to 0.93 for the major ultrastructural features (hypercontracted myofibrils, loss of myofilaments, Z line irregularities, mitochondrial enlargement, and collagen replacement of muscle fibers), indicating substantial to almost perfect agreement between the observers. The observations were recorded descriptively for each specimen, and representative light microscopic and TEM images illustrating the characteristic morphological features were selected for photographic documentation. The frequencies of each histopathological and ultrastructural feature were summarized across the study specimens.

Statistical analysis

As this investigation was designed as a descriptive observational study, no hypothesis testing or inferential statistical analysis was performed. The collected data were entered into statistical software (IBM SPSS, version 22, IBM Corp., Armonk, NY) for data management and descriptive analysis. Categorical variables were summarized as frequencies and percentages, whereas continuous variables were presented as mean ± standard deviation (SD). The microscopic findings were described qualitatively and quantitatively to illustrate the spectrum of histopathological and ultrastructural alterations observed in OSMF, without performing intergroup comparisons or significance testing.

## Results

Ten buccal mucosal biopsy specimens were included in the study, comprising seven specimens from patients diagnosed with OSMF and three specimens obtained from healthy controls. Based on the clinical assessment of maximum mouth opening, 2 (28.6%) OSMF cases were categorized as grade I, 4 (57.1%) as grade II, and 1 (14.3%) as grade III, whereas all control participants demonstrated normal mouth opening greater than 40 mm (Table 1).

**Table 1:** Distribution of clinical grading of mouth opening among the study participants Data are presented as frequency (percentage). OSMF: oral submucous fibrosis

Clinical grade	OSMF (n = 7)	Control (n = 3)
Grade I (>40 mm)	2 (28.6%)	3 (100%)
Grade II (20-39 mm)	4 (57.1%)	0 (0%)
Grade III (<20 mm)	1 (14.3%)	0 (0%)

Histopathological examination of hematoxylin and eosin-stained sections revealed characteristic muscle alterations in OSMF specimens. Muscle fiber atrophy, splitting of muscle fibers, flocculent degeneration, hyaline degeneration, and fibrosis surrounding the muscle bundles were observed in all 7 (100%) OSMF cases. Constricted blood vessels were identified in 5 (71.4%) specimens, whereas vacuolar degeneration and dilated blood vessels were observed in 2 (28.6%) specimens each. None of these alterations were identified in the control specimens, which exhibited normal muscle architecture without degeneration or fibrosis (Table 2).

**Table 2:** Light microscopic features of buccinator muscle alterations Data are presented as frequency (percentage). Findings were evaluated using hematoxylin and eosin-stained tissue sections. OSMF: oral submucous fibrosis

Histopathological feature	OSMF (n = 7)	Control (n = 3)
Muscle fiber atrophy	7 (100%)	0 (0%)
Splitting of muscle fibers	7 (100%)	0 (0%)
Flocculent degeneration	7 (100%)	0 (0%)
Hyaline degeneration	7 (100%)	0 (0%)
Fibrosis around muscle bundles	7 (100%)	0 (0%)
Vacuolar degeneration	2 (28.6%)	0 (0%)
Constricted blood vessels	5 (71.4%)	0 (0%)
Dilated blood vessels	2 (28.6%)	0 (0%)

**Figure 1:**
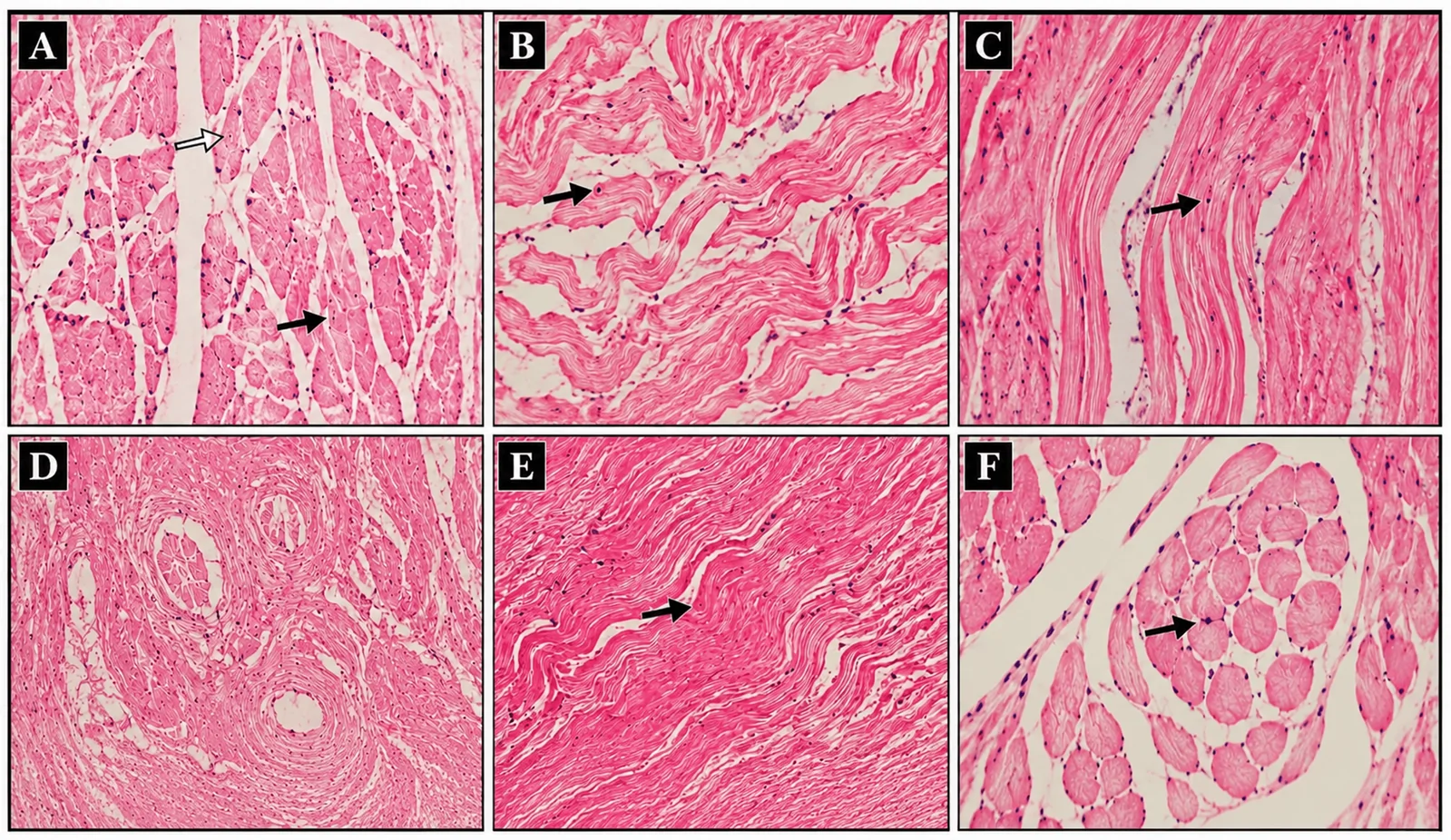
Representative light microscopic features of buccinator muscle alterations in oral submucous fibrosis (hematoxylin and eosin stain, ×400) (A) Atrophic skeletal muscle fibers showing reduced fiber diameter (black arrow) with widened endomysial connective tissue spaces (white hollow arrow). (B) Splitting of skeletal muscle fibers (black arrow). (C) Flocculent degenerative material within split muscle fibers (black arrow). (D) Dense fibrosis surrounding and separating muscle bundles. (E) Hyaline degeneration of skeletal muscle fibers (black arrow). (F) Cross-sectional view of skeletal muscle demonstrating two to three peripherally placed nuclei within individual muscle fibers (black arrow).

Representative photomicrographs demonstrating muscle fiber atrophy and splitting (Figure 1A), hyaline degeneration (Figure 1B), flocculent degeneration (Figure 1C), constricted blood vessels (Figure 1D), fibrosis surrounding muscle bundles (Figure 1E), and vacuolar degeneration (Figure 1F) are presented.

Ultrastructural examination using TEM demonstrated marked degenerative changes in the buccinator muscle fibers of all OSMF specimens. Loss of myofibrils, loss of myofilaments, focal disappearance of I bands and Z lines, irregular Z lines, enlarged mitochondria, enlarged muscle nuclei, autophagic vacuoles, and collagen replacement of muscle fibers were consistently observed in all OSMF specimens. In contrast, the control specimens showed preserved sarcomeric organization, normal mitochondria, intact muscle nuclei, and absence of degenerative ultrastructural changes (Table 3).

**Table 3:** Ultrastructural alterations observed by transmission electron microscopy Data are presented as frequency (percentage). Ultrastructural observations were obtained using transmission electron microscopy. OSMF: oral submucous fibrosis

Ultrastructural feature	OSMF (n = 7)	Control (n = 3)
Loss of myofibrils	7 (100%)	0 (0%)
Loss of myofilaments	7 (100%)	0 (0%)
Focal loss of I bands	7 (100%)	0 (0%)
Focal loss of Z lines	7 (100%)	0 (0%)
Irregular Z lines	7 (100%)	0 (0%)
Enlarged mitochondria	7 (100%)	0 (0%)
Enlarged muscle nuclei	7 (100%)	0 (0%)
Autophagic vacuoles	7 (100%)	0 (0%)
Collagen replacing muscle fibers (fibrosis)	7 (100%)	0 (0%)

**Figure 2:**
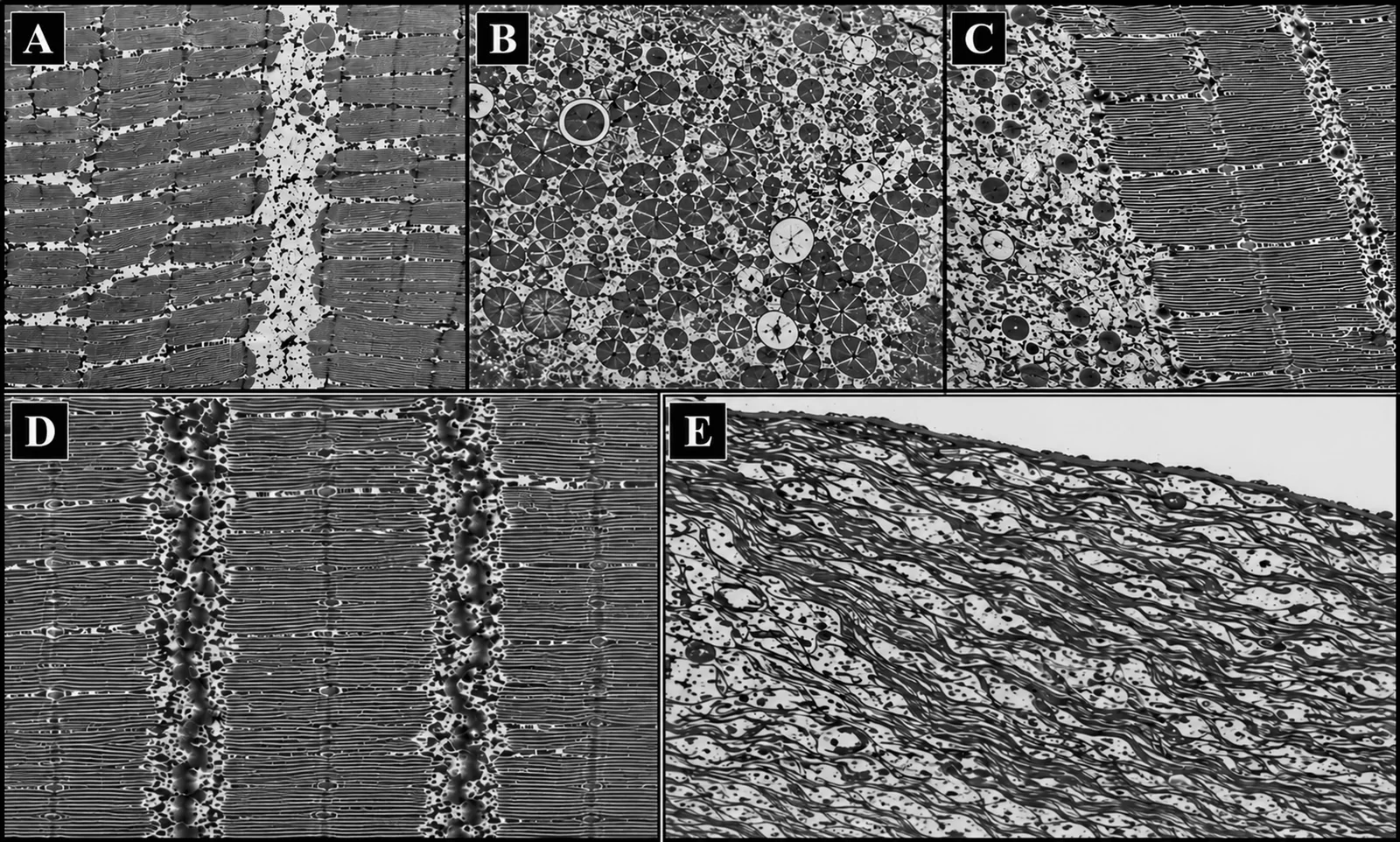
Representative transmission electron micrographs demonstrating ultrastructural alterations of the buccinator muscle in oral submucous fibrosis (A) Splitting of myofibrils (black arrow) with focal loss of myofibrils (black arrow). (B) Replacement of myofibrils by granular degenerative material (black arrow), mitochondrial accumulation (black arrows), and autophagic vacuoles (white circles). (C) Loss of myofilaments resulting in disruption of the contractile apparatus (black arrow). (D) Irregular Z lines indicating sarcomeric disorganization (black arrows). (E) Collagen fibrils replacing degenerating muscle fibers (black arrows) beneath the preserved basal lamina (black arrow).

Representative TEM images depicting splitting of myofibrils with focal loss of myofibrils (Figure 2A), replacement of myofibrils by granular degenerative material with mitochondrial accumulation and autophagic vacuoles (Figure 2B), loss of myofilaments resulting in disruption of the contractile apparatus (Figure 2C), irregular Z lines indicating sarcomeric disorganization (Figure 2D), and collagen fibrils replacing degenerating muscle fibers beneath the preserved basal lamina (Figure 2E) are presented.

Overall, the descriptive histopathological and ultrastructural evaluations demonstrated consistent muscle degeneration and fibrosis in the buccinator muscle of patients with OSMF. Light microscopy identified characteristic tissue-level alterations, whereas TEM provided detailed visualization of intracellular and ultrastructural abnormalities, supporting the presence of progressive muscle involvement in patients with OSMF.

## Discussion

The present descriptive study evaluated the histopathological and ultrastructural alterations of the buccinator muscle in OSMF using light microscopy and TEM. The findings demonstrated consistent muscle degeneration in all OSMF specimens, characterized by muscle fiber atrophy, splitting of muscle fibers, flocculent degeneration, hyaline degeneration, fibrosis, vascular alterations, and characteristic ultrastructural abnormalities, including hypercontracted myofibrils, loss of myofibrils and myofilaments, disruption of I bands and Z lines, enlarged mitochondria, autophagic vacuoles, and collagen replacement of muscle fibers. These observations indicate that muscle degeneration is an integral component of OSMF and may substantially contribute to the progressive limitation of mouth opening in affected patients.

Although subepithelial fibrosis is considered the hallmark of OSMF, increasing evidence suggests that the underlying musculature is equally affected during disease progression [6-8]. Chronic chewing of areca nut produces repetitive mechanical trauma and exposure to alkaloids such as arecoline, resulting in persistent inflammation, oxidative stress, activation of fibroblasts, and excessive collagen deposition [11]. Progressive fibrosis compresses blood vessels, reduces tissue perfusion, and creates a hypoxic microenvironment that promotes the degeneration of adjacent skeletal muscle fibers [6,12]. Recent reviews have highlighted hypoxia, persistent inflammation, and abnormal collagen metabolism as the central mechanisms responsible for disease progression and tissue remodeling in OSMF [1-3].

In the present study, all OSMF specimens exhibited muscle fiber atrophy and fibrosis under light microscopy. These findings support the concept that degeneration of the buccinator muscle is not merely a secondary consequence of reduced oral function but may represent an important pathological component of the disease itself. Long-standing fibrosis surrounding muscle bundles restricts normal muscle contraction, and reduced vascularity further aggravates ischemic injury and muscle wasting. Continuous replacement of muscle fibers by dense collagen tissue gradually decreases muscle elasticity, explaining the progressive reduction in mouth opening that is observed clinically. Similar observations have been reported in histopathological studies demonstrating extensive degeneration of skeletal muscle fibers with increasing OSMF severity [6-8,12].

Gupta et al. [13] emphasized that the pathogenesis of OSMF extends beyond areca nut exposure alone and highlighted the potential role of plasma fibrinogen degradation products as biomarkers reflecting disease activity and progression. These findings support the concept that persistent inflammation and abnormal extracellular matrix remodeling may contribute not only to subepithelial fibrosis but also to progressive degeneration of the underlying musculature observed in the present study.

The vascular changes observed in the present study, particularly constricted blood vessels, are important pathological findings. Progressive collagen accumulation around blood vessels compromises microcirculation, reducing oxygen and nutrient delivery to skeletal muscles [7]. Chronic ischemia promotes mitochondrial dysfunction, oxidative injury, and activation of apoptotic pathways, ultimately leading to muscle tissue degeneration and fibrosis [12,14]. Contemporary molecular studies have emphasized the role of hypoxia-inducible pathways in sustaining fibrosis and promoting disease progression in OSMF, supporting the vascular changes observed in this study [7,15].

TEM provided additional insights into the intracellular changes associated with muscle degeneration. The loss of myofilaments, disruption of sarcomeric architecture, enlarged mitochondria, and autophagic vacuoles observed in all OSMF specimens indicate severe structural damage affecting the contractile apparatus of the skeletal muscle. Mitochondrial enlargement likely represents a response to chronic oxidative stress and impaired cellular respiration, whereas autophagic vacuoles reflect the activation of intracellular degradation pathways attempting to eliminate damaged organelles. The progressive destruction of myofibrils ultimately results in reduced muscle strength and replacement of muscle tissue by collagen fibers. Earlier ultrastructural investigations by El-Labban and Canniff [8] first demonstrated these degenerative changes, while later studies by Sumathi et al. [6] confirmed similar findings in patients with advanced OSMF. A recent systematic review concluded that restricted mouth opening is determined not only by subepithelial fibrosis but also by the extent of muscle degeneration, reinforcing the observations of the present study [7].

Sharma and Radhakrishnan [16] proposed that limited mouth opening results from a combination of submucosal fibrosis, muscle fibrosis, stretch-mediated muscle damage, vascular alterations, and hypoxia-induced metabolic changes. These mechanisms collectively impair muscle elasticity and contractility, contributing to the progression of trismus. The ultrastructural findings of this study also explain why the severity of trismus is often disproportionate to the degree of connective tissue fibrosis. While fibrosis limits tissue elasticity, degeneration of contractile elements further compromises muscle function by reducing force generation and increasing stiffness. Loss of normal sarcomeric organization, together with collagen replacement of muscle fibers, results in irreversible functional impairment. Therefore, the assessment of muscle involvement may provide additional pathological information beyond the conventional evaluation of connective tissue fibrosis alone.

The clinical implications of these findings are significant. Current management strategies for OSMF primarily target inflammation and fibrosis using antioxidants, corticosteroids, hyaluronidase, physiotherapy, and surgical release in advanced disease. However, the present findings suggest that muscle degeneration is another important therapeutic target. Preservation of muscle viability through early diagnosis, habit cessation, physiotherapy, and therapies directed at reducing oxidative stress and improving tissue vascularity may help preserve muscle function before irreversible degeneration occurs. Therefore, future therapeutic approaches should consider both connective tissue fibrosis and skeletal muscle pathology to improve functional outcomes in patients with OSMF.

The present study has several limitations. First, this was an exploratory descriptive investigation involving a small number of biopsy specimens and a small control group. Consequently, the findings should be regarded as preliminary and hypothesis-generating rather than definitive. The control group was not formally matched to the OSMF group for age and sex, and the small sample size limits the generalizability of the observations. The biopsy procedure did not include prospective recording of precise millimetric depth, anatomical coordinates, or standardized mapping of the sampled buccinator subregion. In addition, a predefined number of microscopic fields, muscle fibers, or vessels and a standardized protocol for selecting representative TEM fields were not established. Quantitative morphometric analysis of the histopathological or ultrastructural alterations was not performed, and the findings were assessed qualitatively as present or absent rather than using a standardized semiquantitative or quantitative grading system.

Consequently, the reported frequencies indicate the presence of a particular feature within the examined specimen but do not reflect its extent or severity, and direct correlation between microscopic findings and clinical OSMF grade or mouth opening could not be established. Complete observer blinding to the clinical group could not be ensured because marked morphological differences between control and OSMF specimens could potentially reveal group allocation during microscopic evaluation. Immunohistochemical or molecular evaluation of fibrosis- and hypoxia-related biomarkers was also beyond the scope of this study. Future studies involving larger and appropriately matched cohorts, standardized biopsy site documentation and microscopic sampling protocols, quantitative morphometric analyses, and correlation of ultrastructural findings with molecular markers and clinical severity are warranted to validate and expand upon these observations.

## Conclusions

The present exploratory study demonstrates histopathological and ultrastructural alterations of the buccinator muscle in the OSMF specimens examined, including muscle fiber degeneration, myofibrillar disruption, mitochondrial abnormalities, and collagen replacement. These findings suggest that skeletal muscle alterations may accompany the fibrotic changes of OSMF; however, their temporal relationship, pathogenic significance, and contribution to restricted mouth opening cannot be established from the present cross-sectional descriptive design. Larger studies incorporating quantitative morphometric assessment and clinical-histopathological correlation are warranted to validate these observations and clarify the role of muscle involvement in OSMF.
